# Phenotypic Variability of Kidney Involvement in Fabry Disease—Lessons from a Family Study

**DOI:** 10.3390/life16060866

**Published:** 2026-05-22

**Authors:** Elena-Emanuela Rusu, Ruxandra-Oana Jurcut, Mihaela Gherghiceanu, Filip Muresan, Gheona Altarescu, Bogdan Stanciulescu, Robert Adam, Alexandru Procop, Cristina Stoica, Bogdan Marian Sorohan, Vlad Stefanescu, Gener Ismail

**Affiliations:** 1 Department of Nephrology, “Carol Davila” University of Medicine and Pharmacy, 020021 Bucharest, Romania; 2 Department of Nephrology, Fundeni Clinical Institute, 022328 Bucharest, Romania; 3 Department of Cardiology, “Carol Davila” University of Medicine and Pharmacy, 020021 Bucharest, Romania; rjurcut@gmail.com (R.-O.J.);; 4 Department of Cardiology, Emergency Institute for Cardiovascular Diseases “Prof. Dr. C. C. Iliescu”, 022328 Bucharest, Romania; 5 Department of Cell Biology and Histology, “Carol Davila” University of Medicine and Pharmacy, 020021 Bucharest, Romania; 6 “Victor Babes” National Institute for Research and Development in Pathology and Biomedical Sciences, 050097 Bucharest, Romania; 7 Shaare Zedek Institute of Medical Genetics, Shaare Zedek Medical Center, Shmu’el Bait St 12, Jerusalem 9103102, Israel; 8 Anatomic Pathology, Fundeni Clinical Institute, 022328 Bucharest, Romania; 9 Department of Pediatric Nephrology, Fundeni Clinical Institute, 022328 Bucharest, Romania; 10 Department of Uronephrology and Kidney Transplantation, Fundeni Clinical Institute, 022328 Bucharest, Romania; 11 Department of Neurology, Fundeni Clinical Institute, 022328 Bucharest, Romania

**Keywords:** Fabry disease, family screening, kidney involvement, kidney biopsy

## Abstract

Fabry disease is an X-linked lysosomal storage disease that leads to the intracellular accumulation of glycosphingolipids in many tissues and fluids, including the kidneys. We report a single family with Fabry disease that includes seven patients carrying the pathogenic variant c.797A>C in the *GLA* gene, with remarkable variability in kidney involvement, assessed based on clinical, biological, and histological data. The patients were monitored for 2–9 years, and all received enzyme replacement therapy. Kidney involvement was variable and included severely decreased GFR with significant proteinuria, mildly to moderately decreased GFR with proteinuria, mildly decreased GFR with microalbuminuria or normoalbuminuria, hyperfiltration with normoalbuminuria, and preserved kidney function. All patients who underwent kidney biopsy presented with Fabry-specific lesions and, in some cases, chronic histological damage. This study provides valuable insights into kidney involvement evaluated through kidney biopsy, personalized management strategies for family members according to their phenotype, and long-term follow-up of kidney function. We underscore the importance of molecular screening of the *GLA* gene in all family members for early identification of the disease and early initiation of specific treatments that can prevent or delay the progression of this disease.

## 1. Introduction

Fabry disease (FD) is a systemic, rare, X-linked, recessive lysosomal storage disease caused by pathogenic variants in the α-galactosidase A (GLA) gene; the disease leads to severe organ damage, including kidney failure, hypertrophic cardiomyopathy, and stroke [1,2]. Diagnostic delays are attributed to phenotype variability, disease rarity, and the non-specificity of early symptoms [3].

Nephropathy is one of the major complications of this disease [4]. Untreated classically affected males develop proteinuria and progressive renal impairment in the second to third decades of life and typically progress to kidney failure by the fourth to fifth decades of life [2,4,5]. In women, because of random X-chromosomal inactivation, the disease progression is variable, ranging from nonprogressive asymptomatic carriers to a classic phenotype, with symptoms as severe as those of men [6,7]. Clinical diagnosis of Fabry nephropathy relies on a glomerular filtration rate (GFR) < 90 mL/min/1.73 m^2^, albuminuria > 30 mg/g creatinine, and proteinuria > 300 mg/day or >300 mg/g creatinine, after the exclusion of other kidney diseases [5]. Morphologic studies have demonstrated that kidney lesions are present even before clinical signs of nephropathy become visible [8,9,10,11,12,13]. As corresponding histological abnormalities, pathological albuminuria and proteinuria include globotriaosylceramide (GL-3) accumulation in podocytes. At the ultrastructural level, podocyte injury and foot process effacement precede pathological albuminuria [2,8,9,10,11,12,13,14,15]. GL-3 storage can also be observed in various kidney cell types: distal tubules, epithelium of the loop of Henle, and endothelial and smooth muscle cells of the renal arterioles [2,11]. Impaired kidney function with a progressive decrease in the glomerular filtration rate occurs in patients with chronic histological lesions, such as glomerulosclerosis, tubular atrophy, interstitial fibrosis, microvascular endothelial GL-3 accumulation, and arteriolar injury [5,11,14]. Kidney biopsies can be a very important diagnostic tool for assessing renal involvement and excluding the coexistence of other kidney diseases [2,5,13,16,17]. Kidney involvement in Fabry disease has significant variability between patients, even in those with the same pathogenic GLA gene variant from the same family [18,19,20,21,22,23,24].

Here, we report a case series of seven patients belonging to the same family, aiming to assess the renal phenotype via clinical, biological, and pathological data. We highlight the remarkable variability of the renal phenotype within the family, the importance of appropriate family screening, and the assessment of kidney impairment to establish indications for specific treatment initiation. We also report the long-term follow-up of the family members, showing disease progression and demonstrating the importance of timely treatment initiation. Between 2017 and 2026, all patients were monitored by our Fabry multidisciplinary team. Between diagnosis and the latest evaluation, the follow-up ranged from 2 to 9 years.

## 2. Materials and Methods

### 2.1. Study Design

We report a case series of 7 patients from a single family carrying the pathogenic variant c.797A>C in the *GLA* gene. Our main aim was to assess kidney involvement based on clinical, biological, and histological data; the implications of kidney evaluation on patient management; and the long-term evolution of kidney function.

**Figure 1:**
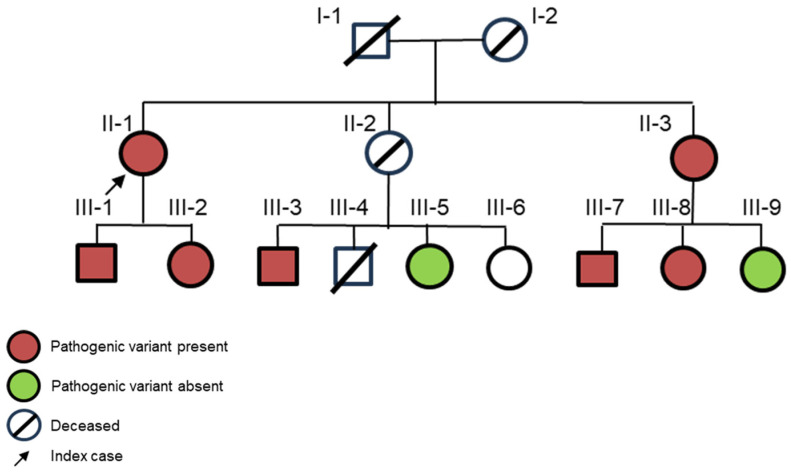
Family pedigree showing the index case—a heterozygous female (II-1)—and the family screening results. Molecular analysis of the GLA gene showed the c.797A>C variant in 7 patients from two generations. The index patient is marked with an arrow. Both parents of the index case were already deceased (I-1, I-2), both at 73 years old, with the presumed causes of death being liver cirrhosis for the father and pulmonary cancer for the mother. The proband’s son (III-1) and daughter (III-2) had the pathogenic variant in the GLA gene. The second branch of the family included the proband’s sister (II-2), with kidney failure requiring dialysis. She was deceased and lacked an FD diagnosis, but based on her clinical manifestation and X-linked transmission of the disease, we presume she had it. Patient II-2 had two sons, one (deceased) with kidney failure requiring hemodialysis and the other with CKD and diagnosed with FD. Cascade genotyping of GLA in the index case sister (II-3) and her children revealed another 3 affected family members.

Seven patients (3 males and 4 females) from the same family spanning two generations (Figure 1) were retrospectively investigated in this study; they were comprehensively evaluated by the multidisciplinary team of the Expert Center for Rare Disease of the Fundeni Clinical Institute, Bucharest, and the Emergency Institute for Cardiovascular Diseases “Prof. Dr. C. C. Iliescu”, Bucharest. At baseline and over a follow-up period of up to 9 years, we collected data regarding demographics; clinical, biological, histological, and molecular data; clinical events; and comorbidities.

### 2.2. Clinical Assessments

The evaluation of kidney involvement included measuring serum creatinine and the estimated glomerular filtration rate (eGFR) (with eGFR being estimated using the 2021 CKD Epidemiology Collaboration creatinine equation [25] and with the Schwartz formula for children [26]). We also measured the urine albumin:creatinine ratio (UACR) in spontaneous urine and 24 h proteinuria. Kidney biopsies were also performed. Chronic kidney disease (CKD) is classified based on GFR category: G1, normal/high (GFR ≥ 90 mL/min/1.73 m^2^); G2, mildly decreased (89–60 mL/min/1.73 m^2^); G3a, mildly to moderately decreased (45–59 mL/min/1.73 m^2^); G3b, moderately to severely decreased (30–44 mL/min/1.73 m^2^); G4, severely decreased (15–29 mL/min/1.73 m^2^); G5, kidney failure (<15 mL/min/1.73 m^2^) [27]. Albuminuria and proteinuria were used as biomarkers for FD-related nephropathy. Albuminuria was expressed as a ratio to urinary creatinine, and proteinuria was determined from a 24 h urinary volume. A urinary albumin:creatinine ratio (UACR) < 30 mg albumin/g creatinine was considered normal to mildly increased, a UACR of 30–300 mg/g was classified as moderately increased, and a UACR > 300 mg/g was considered severely increased [27]. GAL-A enzyme activity and Lyso-GL-3 levels were determined, and genetic analysis was performed on plasma using dried blood spots on filter paper at the medical laboratories of ARCHIMED Life Science GmbH (ARCHIMEDlife: www.archimedlife.com, accessed on 11 April 2026).

Kidney biopsy was performed for the following reasons:

– To quantify GL-3 deposits and chronic lesions (glomerulosclerosis and interstitial fibrosis);
– To assess the presence of significant renal deposits in women without evidence of FD nephropathy and to establish indications to initiate FD-specific therapy;
– To exclude coexistent kidney diseases;
– To assess the kidney damage in patients with associated diseases affecting the kidneys.

Kidney biopsy specimens were studied via light microscopy and electron microscopy. 

### 2.3. Follow-Up Protocol

Patients were monitored annually if the risk of disease progression was low, every 6 months if the risk was moderate, and every 3 months if the risk was very high, according to CKD prognosis based on eGFR and albuminuria categories [14,28].

## 3. Results

### Patients

**Figure 2:**
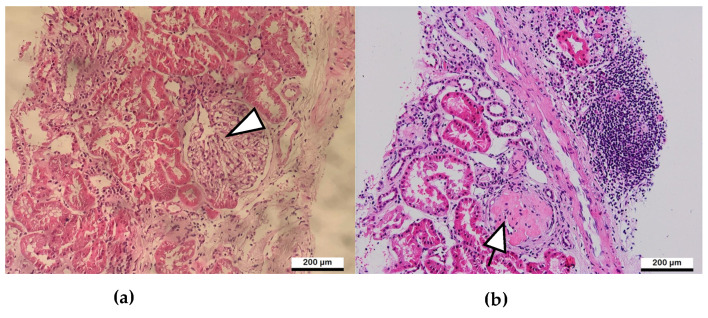
Light microscopy of patient II-1 (hematoxylin and eosin staining; scale bar: 200 µm): (**a**) cortical renal parenchyma showing segmental vacuolated appearance of podocytes (arrowhead); (**b**) global glomerulosclerosis (arrow).

The family index case (II-1) is a 49-year-old female with a history of hypertrophic cardiomyopathy and a stroke at the age of 43. Six years after the first clinical manifestations, she was diagnosed with Fabry disease by a cardiologist based on clinical phenotype and genetic testing. Molecular analysis identified a pathogenic missense classic GLA variant (c.797A>C; p.Asp266Ala). Specific laboratory tests for FD found borderline α-galactosidase levels at 1.2 μmol/L/h (cut-off > 1.2) and mildly increased lyso-GL-3 at 6.7 ng/mL (cut-off < 3.5) (Table 1). Cardiological workup revealed a short PR interval on electrocardiogram and increased left ventricular (LV) voltage; severe biventricular hypertrophic cardiomyopathy; and preserved LV ejection fraction. She also presented with perioral angiokeratoma at the skin level, acroparesthesia, white matter lesions, and chronic lacunar ischemic lesions as neurological manifestations. Nephrological evaluation showed microalbuminuria, proteinuria, and mildly to moderately decreased eGFR, corresponding to CKD stage G3a (Table 1). Ultrasound showed a decrease in kidney size and parenchyma. A kidney biopsy was performed to broaden the evaluation, and it showed specific lesions and chronic lesions (Table 2). Vacuoles were frequently observed in podocytes, tubules, and endothelial cells, corresponding to extracted GL-3 deposits (Figure 2a). Segmental and global glomerulosclerosis (Figure 2b), moderate interstitial fibrosis, tubular atrophy, and vasculopathy were also observed.

**Figure 3:**
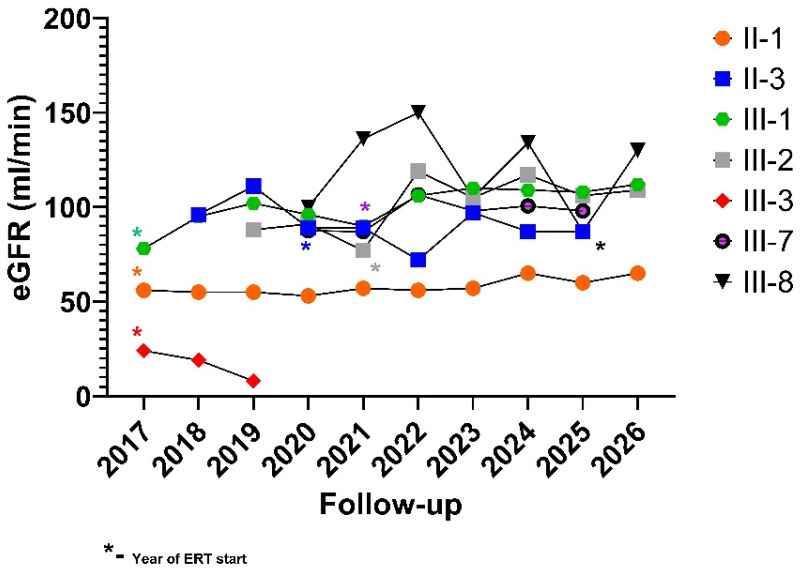
Evolution of GFR during the follow-up period for family members. The year of treatment initiation is marked by an asterisk.

Therapeutic management comprised an angiotensin receptor inhibitor to decrease proteinuria and enzyme replacement therapy (ERT) with agalsidase beta at a dose of 1 mg/kgc every 2 weeks. Kidney function stabilized and subsequently slowly improved (Figure 3). From a cardiological point of view, the patient remained symptomatic under treatment; she subsequently underwent surgical septal myectomy and mitral valvuloplasty, with favorable evolution [29].

Molecular, clinical, and biochemical tests were extended to the proband’s family members. The family pedigree is shown in Figure 1. Six additional affected family members were identified. Fabry disease-specific features, renal manifestations at baseline, kidney biopsy, and the family’s extrarenal phenotype are presented in Table 1.

**Figure 4:**
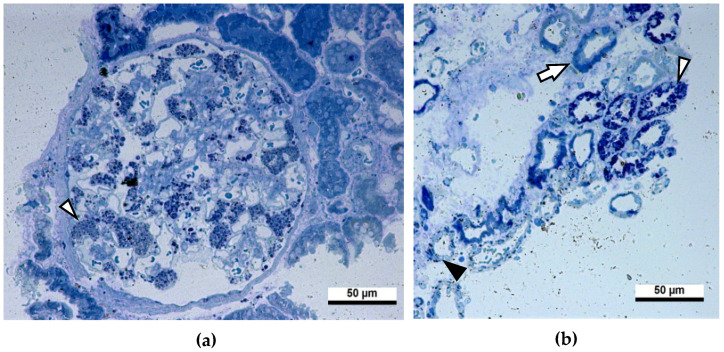
Light microscopy of the patient III-1 (toluidine blue staining of resin-embedded section, scale bar 50 µm). (**a**) Glomerulus with podocyte densely stained glycosphingolipids and lysosomal accumulations—arrowhead. Most podocytes seem to be affected. (**b**) Glycosphingolipid deposits can be seen in the smooth muscle cells of the arterioles (black arrowhead) and distal tubules (arrowhead), while proximal tubules remain mostly unaffected by the disease (arrow).

**Figure 5:**
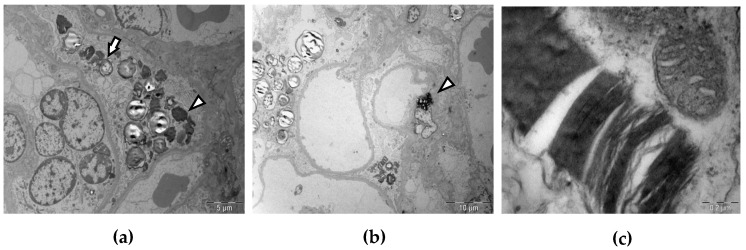
Electron micrograph images from patient III-1. (**a**) A podocyte with characteristic laminated glycosphingolipid lysosomal inclusions (“zebra bodies”)—arrow. Some deposits may be homogeneous, without a particular substructure—arrowhead; scale bar: 5 µm. (**b**) Some mesangial cells also presented with cytoplasmic lipid inclusions—arrowheads; scale bar: 10 µm. (**c**) Characteristic laminated structure of glycosphingolipid inclusions; scale bar: 0.2 µm.

The index patient had one son and one daughter. The proband’s 29-year-old son (number III-1) had the GLA familial variant; α-GLA activity was 0.0 µmol/L/h, and lyso-GL-3 was 129.2 ng/mL. He presented with acroparesthesia, angiokeratomas, hypohidrosis, CKD stage G2 (eGFR = 78 mL/min/1.73 m^2^), and proteinuria (240 mg/day). He also had cornea verticillata, a normal brain magnetic resonance imaging (MRI) exam, and a normal cardiological evaluation (ECG, transthoracic echocardiography, and cardiac MRI). A kidney biopsy (Table 2) specimen was evaluated using light microscopy and showed typical GL3 accumulation in the podocytes, distal tubular epithelium (Figure 4a,b), mesangium, and parietal epithelium. Arteries showed deposits in endothelial and smooth muscle cells (Figure 4b). Electron microscopy (EM) revealed numerous zebra bodies within the podocyte cytoplasm in the glomerulus (Figure 5a–c). Under EM, we observed segmental podocyte foot process effacement.

The sister of the index patient (II-2) had kidney failure requiring dialysis and died at the age of 54. One of her sons had kidney failure, underwent hemodialysis, and died at 28 years old. Her second son, patient number III-3, was identified with the familial genotype at 29 years old and had α-GLA activity of 0.0 µmol/L/h and lyso-GL-3 of 68.2 ng/mL. He had CKD stage G3b for 2 years, Fabry disease with severe CKD (corresponding to stage G4), and secondary arterial hypertension (Table 1). Due to advanced CKD, we did not perform a kidney biopsy on this patient. Multidisciplinary evaluation also revealed other target organ impairments: left ventricular hypertrophy, acroparesthesia, white matter lesions on brain MRI, hypohidrosis, and cornea verticillata.

The two related young male patients, patient III-1 and patient III-3, had the same age at diagnosis (29 years) but had remarkably different disease severity, as well as differing disease evolution and life trajectory. Following diagnosis and complete evaluation, both patients started ERT. Patient III-1 received ERT when he presented with mildly decreased eGFR and mild proteinuria. After nine years of follow-up, his general condition and kidney function were excellent (Figure 3). For his cousin of the same age, patient III-3, ERT was initiated after he had already presented with advanced organ damage. Eight months after the diagnosis, despite the initiation of ERT, kidney function rapidly deteriorated, and he required hemodialysis. Unfortunately, after 2 years of hemodialysis, he died at home from sudden cardiac arrest. In his case, the treatment was ineffective for kidney impairment but prevented left ventricular hypertrophy progression and other target organ damage, thus maintaining his eligibility for kidney transplantation.

Upon family screening, another two females and two children were diagnosed with the familial genetic variant. The females were the proband′s daughter (III-2) and sister (II-3).

**Figure 6:**
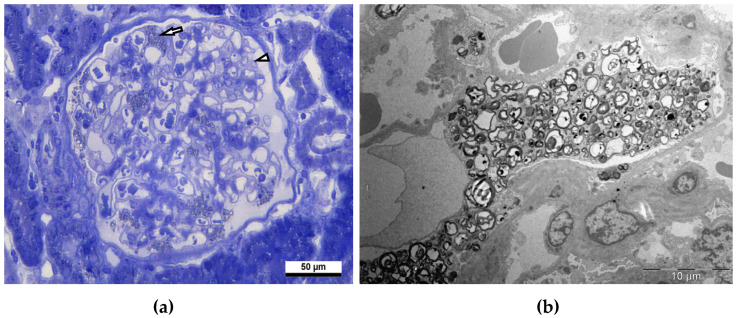
Light microscopy aspect of resin-embedded tissue sections (toluidine blue) (panel a; scale bar: 50 µm) and electron micrograph (panel b; scale bar: 10 µm) from patient III-2, a heterozygous female. (**a**) Glomerulus demonstrating cellular mosaicism due to random X-chromosome inactivation. Note the heterogeneous population of podocytes, with some showing heavy inclusions (arrow) and others appearing normal (arrowhead). (**b**) Podocyte with characteristic lipid cytoplasmic inclusions.

The proband′s daughter, patient III-2, carried the heterozygous GLA c.797A>C variant and had decreased α-Gal A activity (0.7 µmol/L/h) and slightly increased lyso-GL-3 (3.9 ng/mL). The multidisciplinary clinical evaluation showed acroparesthesia and angiokeratomas on the lips and cornea verticillata; however, she had a normal heart structure and function, a normal T1 value on cardiac MRI, and a normal cerebral MRI. Nephrological evaluation showed mildly decreased eGFR (eGFR 88 mL/min/1.73 m^2^) and 24 h urinary protein excretion of 200 mg, without albuminuria. To better evaluate kidney involvement, a kidney biopsy was performed. Despite mild biological abnormality, the kidney histology showed numerous lysosomes with lamellated contents in podocytes (Figure 6a,b), mesangial cells, parietal epithelial cells, vascular smooth muscle cells, tubular epithelia of the distal tubules, and podocyte foot effacement in the affected podocytes. In this heterozygous female, the podocytes were heterogeneously affected by the disease due to the random inactivation of the X chromosome during embryogenesis (Figure 6a). At that time, the clinical and biological data from the initial evaluation did not meet the criteria for ERT according to the Romanian national protocol criteria, and annual multidisciplinary follow-up was recommended. We noticed a decline in eGFR during follow-up. Based on the decreasing trend in renal function, the histological findings, and given the family history, FD-specific therapy was approved, although the specific criteria of the Romanian protocol at that time were still not fulfilled. She started agalsidase beta two years after FD diagnosis, and her kidney function remained stable over 5 years of follow-up (Figure 3).

**Figure 7:**
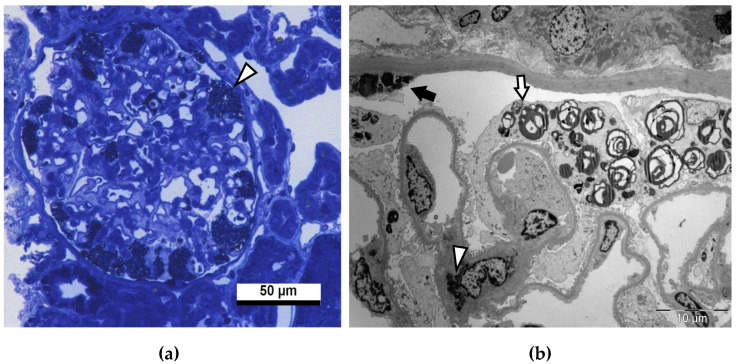
Light microscopy aspect of resin-embedded tissue sections (toluidine blue) (panel a; scale bar: 50 µm) and electron micrograph (panel b; scale bar: 10 µm) from patient II-3. (**a**) Glomerulus of patient II-3 demonstrating focal, densely stained lysosomal glycosphingolipid accumulations within some podocytes (arrowhead). (**b**) Electron microscopy image showing electron-dense lamellate inclusions in the cytoplasm of podocytes (arrow) and glomerular mesangial cells (arrowhead).

Patient II-3, the 45-year-old sister of the index case, had the familial variant, low α-GLA activity (0.4 µmol/L/h), and increased plasma lyso-GL-3 (10.7 ng/mL). Clinically, she presented with fatigue, vertigo, hearing disorders, and acroparesthesia. Cardiological evaluation showed mild mitral regurgitation and mild tricuspid regurgitation with a spontaneous tendency toward bradycardia. Neurological evaluation revealed sensitive polyneuropathy and a hypohidrosis, transient ischemic attack, and chronic vertigo, while brain MRI showed cerebral small vessel disease. Nephrological examination identified CKD stage G1, without albuminuria or proteinuria, but our experience showed that the standard assessment cannot rule out kidney involvement in female patients with FD [13]. We thus performed a kidney biopsy, which revealed numerous lysosomal inclusions in podocytes (Figure 7a,b), vascular smooth muscle cells, distal tubules, and parietal epithelial cells.

Patient II-3 had two children, who were both diagnosed with the *GLA* familial variant: a 9-year-old son (III-7) and a 17-year-old daughter (III-8).

**Figure 8:**
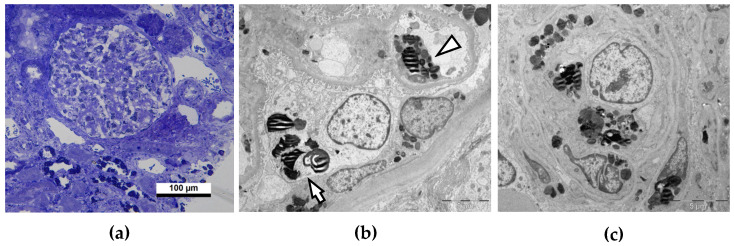
Kidney biopsy findings from the 9-year-old child (patient III-7). (**a**) Glomerulus and tubules with lipid deposits (light microscopy, toluidine blue staining of resin-embedded section; scale bar: 100 µm). (**b**) Podocyte (arrow) and glomerular endothelial cell (arrowhead) with lipid cytoplasmic inclusions (electron microscopy; scale bar: 5 µm). (**c**) Arteriole with lipid accumulation in smooth muscle cells (electron microscopy; scale bar: 5 µm).

The 9-year-old boy (III-7) presented with undetectable α-GLA activity and severely increased plasma lyso-GL-3 (101.1 ng/mL). As a concomitant pathology, he had type 1 diabetes mellitus from the age of 4. He was evaluated at the Department of Pediatric Nephrology and diagnosed with mildly decreased GFR (eGFR = 87.5 mL/min/1.73 m^2^), without albuminuria. We performed a kidney biopsy to assess kidney involvement in Fabry disease and to determine if there were superimposed lesions caused by diabetes. Kidney biopsy showed lamellar inclusions in the podocytes of all glomeruli (Figure 8a–c) and foot process effacement. Additionally, numerous zebra bodies were visible in the endothelium, vascular smooth muscle cells, and in the epithelia of the distal tubules. Cardiological evaluation showed mild left ventricular hypertrophy at transthoracic echocardiography and a low native T1 value (880–920 ms) on cardiac MRI. Neurological evaluation revealed acroparesthesia, but the brain MRI was normal. Due to multiple target organ involvement, ERT treatment was initiated at age 10. Kidney function evolution was favorable over the 5 years of follow-up (Figure 3).

**Figure 9:**
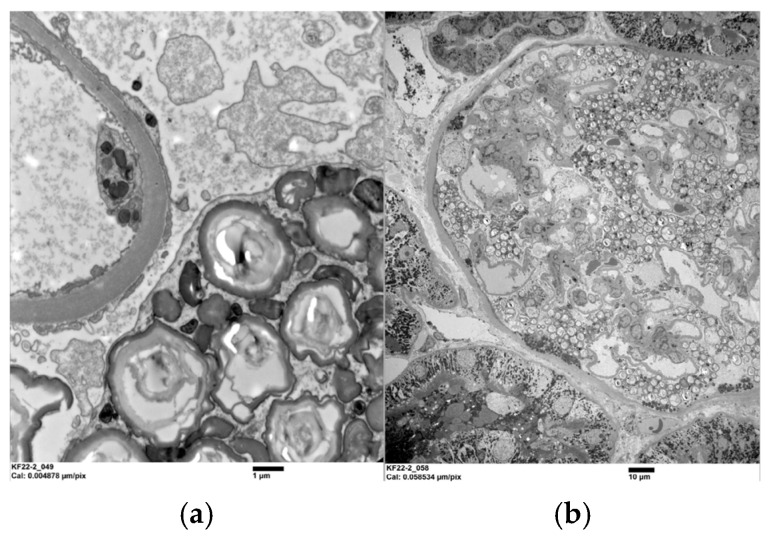
Electron micrograph images from patient III-8 showing lamellar cytoplasmic inclusions in podocytes (panel (**a**);—scale bar: 10 µm; panel (**b**)—scale bar: 1 µm).

The 17-year-old female (patient III-8) was initially evaluated in the pediatric nephrology department and had high GFR (hyperfiltration) with normoalbuminuria and cornea verticillata, without other signs of target organ involvement. After the age of 18, she was monitored at our clinic, and we observed intermittent microalbuminuria. We deepened her assessment by performing a kidney biopsy, which revealed numerous GL3 inclusions in the podocytes, without uniform distribution of GL3 inclusions in podocytes (Figure 9a,b). Segmental effacement of podocyte foot processes and rare segmental detachment were also observed. Numerous GL3 inclusions were present in the parietal epithelial cells and arterial smooth muscle cells. Occasional GL3 inclusions were present in the endothelial cells, and rare GL3 inclusions were present in the mesangial cells and peritubular capillaries. At that time, she did not meet the criteria for ERT according to the Romanian national protocol criteria, and an annual follow-up was recommended. She was monitored through clinical, biological, and imaging data, as well as biomarker evolution. We observed persistent microalbuminuria and a decrease in eGFR over the 4 years of follow-up. The disease further manifested through pain in the extremities and hearing loss, while lyso-GL-3 progressively increased. She was started on ERT (agalsidase alfa) at age 22.

## 4. Discussion

We present a large family diagnosed with Fabry disease, demonstrating significant phenotypic differences among members. Our findings are consistent with previous data from the literature reporting the high variability of organ involvement in patients from the same families [18,19,20,21,22,23,24]. Remarkable variability in renal disease was documented in a large Slovenian family with FD, in which some members had kidney failure, and some had normal kidney function but presented with proteinuria [18]. In addition, extreme variability in disease manifestations was observed in an extended Italian family, emphasizing the need to consider other factors involved in the pathogenesis of FD [20]. Rigoldi et al. showed a high degree of intrafamilial phenotypic variability and underscored the difficulty in making an accurate prognosis for young people based on their family history [21]. Genotype–phenotype correlations lack consistency and predictive power. In our case report, the same pathogenic *GLA* variant had notably variable phenotypes in terms of target organs and variability in renal manifestations related to age and gender. The proband had a severe cardiac manifestation and moderate kidney damage; her son and daughter had mild kidney involvement; one sister and her two sons had severe kidney disease and needed dialysis; and her second sister experienced preponderant neurologic involvement. Family members had varying kidney involvement, ranging from subclinical to severe. Family screening identified four young patients in early disease stages (two young adults, one teenager, and one child), but one young adult was diagnosed in an advanced stage of the disease, with severe kidney involvement and hypertrophic cardiomyopathy.

We show that cascade screening does not always identify patients in the early stages of kidney involvement, despite their young age. The reported family also included two young male cousins of the same age. When comparing their disease extension at diagnosis, one male had mild kidney involvement, low T1 on cardiac MRI, no enzymatic activity, and high lyso-GL-3, while the other had severe kidney disease, hypertrophic cardiomyopathy, no enzymatic activity, and high lyso-GL-3. Both male patients had the same genotype and no enzymatic α-GAL A activity, suggesting that other genetic and non-genetic modifiers could influence the clinical phenotype. In addition, their disease progression under ERT was radically different. The male with mild kidney involvement had normal kidney function after 9 years of ERT, whereas the second rapidly progressed to kidney failure and ultimately died.

According to the Fabry International Registry data, the progression of kidney disease under ERT is related to its severity before treatment. The indicators for poor renal prognosis are a urinary protein/creatinine ratio above 0.5 g/g and ≥50% sclerotic glomeruli at baseline [30]. Our 29-year-old patient, who rapidly progressed to kidney failure, had increased proteinuria and severe chronic disease at diagnosis, showing that early diagnosis is crucial for the response to ERT.

Fabry disease is a multisystemic disease that requires a high index of suspicion, as the phenotype can be classical or atypical, and family history can reveal other affected organs. The index case from our family had cardiac involvement, but her family history showed kidney involvement. The diagnostic approach should consider any organ involvement and needs a good understanding of early biomarkers and phenotypic heterogeneity. Diagnostic delay is common due to the non-specificity of disease manifestations and the rarity of the disease. Family genetic testing was an effective strategy to identify affected members, most of whom were younger and in earlier stages of the disease than the proband. Early diagnosis of members of the studied family after cascade screening led to the best outcomes.

In this family, females developed renal manifestations later than males. Female patients also showed a wide spectrum of disease expression, ranging from severe Fabry disease manifestations—including kidney failure and advanced cardiac involvement—to subclinical organ involvement. Predicting the clinical phenotype in females remains particularly challenging. Phenotypic variability in heterozygous females is influenced by skewed X-chromosome inactivation, residual α-galactosidase A activity, and age. Current treatment guidelines recommend initiating disease-specific therapy in females once evidence of target organ involvement is demonstrated [14,31]. Therefore, careful evaluation by a multidisciplinary clinical team is essential. In our cohort, renal involvement among female patients with Fabry disease ranged from normal kidney function to mildly to moderately decreased. These findings suggest that standard renal assessment based solely on clinical and laboratory parameters cannot reliably exclude kidney involvement in female patients. Kidney biopsies could be a valuable diagnostic tool for detecting early renal involvement and provide important support for therapeutic decision-making.

Several groups have shown that kidney biopsies are safe as part of baseline FD nephropathy evaluation, providing valuable insights into the severity of kidney involvement. This procedure can reveal both specific GL-3 inclusions and irreversible glomerulosclerosis and fibrosis, which indicate chronicity status, with important prognostic implications [9,10,11,12,13,14,15,16]. We now have a better understanding of the importance of early specific treatment initiation in preventing organ damage in later life and improving mortality and morbidity; thus, over the past decade, the aim of FD-specific therapy has changed from treating organ impairment to prevention and preserving organ function [14,32,33]. The updated recommendations and consensus among global experts have focused on the early indicators of disease progression, including kidney histology [14,33]. Kidney biopsies should be considered in select pediatric cases and in females with non-classical phenotypes to assess disease burden, as well as to decide on specific therapy initiation [14,32]. Performing a kidney biopsy is also important to rule out a second renal disease and to interpret the pathogenicity of GLA variants of unknown significance [14,17].

Furthermore, this report documents the clinical and histological findings within this family and the longitudinal evolution of kidney function over a follow-up ranging from 2 to 9 years. This longitudinal assessment offers insight into renal outcomes across different degrees of kidney involvement and at various stages of ERT initiation. Renal manifestations included severely decreased GFR with significant proteinuria (III-3), mildly to moderately decreased GFR with proteinuria (II-1), mildly decreased GFR with microalbuminuria/proteinuria (III-1, III-2) or normoalbuminuria (III-7), hyperfiltration with normoalbuminuria (III-8), and preserved kidney function (II-3). Notably, all patients who underwent kidney biopsy exhibited Fabry-specific lesions and, in some cases, chronic histological damage (II-1, III-1). Except for the patient who presented with advanced renal impairment at diagnosis, all others demonstrated a favorable renal course after ERT initiation.

Despite recent advances in understanding the molecular mechanisms of Fabry disease, including contributions from genetics and transcriptomics, significant knowledge gaps remain regarding intrafamilial phenotypic variability, optimal use of biomarkers for diagnosis, and the identification of prognostic markers to guide treatment initiation. The underlying causes of phenotypic heterogeneity within the same family continue to be investigated. Emerging evidence indicates that DNA methylation and genetic modifiers play a key role in modulating disease expression in Fabry disease [34].

Altarescu et al. performed gene expression analysis to determine whether non-GLA genes and pathways are influencing the phenotype of patients with the same genetic alteration from our reported family. Preliminary data from this family suggested that non-*GLA* genes and related pathways may influence phenotypic variability. Differentially expressed gene (DEG) analysis identified subsets of genes that were significantly up-regulated or down-regulated in both male and female patients with Fabry disease. Functional analysis revealed that these DEGs are involved in renal, vascular, and cardiac function, as well as sphingolipid metabolism, but transcriptomic profiling has been proposed as an additional tool to improve the interpretation of variable phenotypes in FD patients from the same family [35].

Transcriptome analysis can identify early changes in gene expression associated with the development and progression of Fabry nephropathy [36]. A recent transcriptomic study examining glomerular, tubular, and small arterial gene expression using RNA sequencing demonstrated that, with a few exceptions—particularly in arterial tissue—early initiation of ERT in patients with classical Fabry disease can result in sustained normalization of nephropathy-related gene expression patterns [37]. Further studies are warranted to deepen our understanding of gene expression profiles and their contribution to intrafamilial phenotypic variability.

## 5. Conclusions

Our findings from a single family highlight the phenotypic variability of kidney involvement in patients with the same *GLA* variant of FD. Genetic testing of the entire family was an effective method for identifying other members affected by Fabry disease and enabling early diagnosis. This study provides personalized management strategies and valuable insights into kidney involvement in the same family, as well as the long-term evolution of kidney function. We underline the importance of kidney biopsy in detecting early kidney manifestations and guiding therapeutic decisions, potentially improving long-term outcomes.

## Figures and Tables

**Table 1:** Disease features, renal involvement, kidney biopsy findings, and extrarenal phenotype for the family members with Fabry disease.

	N	II-1	II-3	III-1	III-2	III-3	III-7	III-8
	Gender	Female	Female	Male	Female	Male	Male	Female
Fabry features	Age at onset, years	43	45	12	28		9	17
	Age at genetic test, years	49	45	29	30	29	9	17
	α-GLA activity (µmol/L/h)	1.2 *	0.4	0.0	0.7	0.0	0.0	0.5
	Lyso-GL-3 (ng/mL)	6.7	10.7	129.2	3.9	68.2	101.1	4.4
	Total MSSI at baseline, points	37	14	19	10	30	9	3
Renal manifestations	eGFR, mL/min/1.73 m^2^	56	96	78	88	24	87.5	135
	CKD stage	G3a	G1	G2	G2	G4	G2	G1
	UACR (mg/g)	100	10	30	10	300	10	10
	Proteinuria (g/24 h)	0.4	0.1	0.2	0.2	1.7	0.08	0.08
	Hypertension	Yes	No	No	No	Yes	No	No
	Hypertrophic cardiomyopathy	Yes	No	No	No	Yes	Mild LVH	No
	Cardiac MRI changes	HCM	No	No	No	NA	low T1	No
Extrarenal phenotype at baseline	Hypohidrosis	Yes	Yes	Yes	Yes	Yes	No	No
	Acroparesthesia	Yes	Yes	Yes	Yes	Yes	Yes	No
	Cornea verticillata	Yes	Yes	Yes	Yes	Yes	NA	Yes
	Cerebral MRI changes	Yes	Yes	No	No	No	No	No
	Angiokeratoma	Yes	Yes	Yes	No	Yes	Yes	No

α-GLA, α-galactosidase A; α-GLA activity cut-off value > 2.8 µmol/L/h; * α-GLA activity cut-off value > 1.2 µmol/L/h in patient II-1; lyso-GL-3, globotriaosylsphingosine; lyso-GL-3 (ng/mL) cut-off value < 3.5 ng/mL; eGFR, estimated glomerular filtration rate; CKD, chronic kidney disease; UACR, urine albumin/creatinine ratio; NA, not available; MRI, magnetic resonance imaging; LVH, left ventricular hypertrophy; HCM, hypertrophic cardiomyopathy.

**Table 2:** **Histological** **findings.**

	N	II-1	II-3	III-1	III-2	III-3	III-7	III-8
	Gender	Female	Female	Male	Female	Male	Male	Female
Kidney biopsy	Age at kidney biopsy	50	46	29	30	NA	9	19
	Podocyte GL-3 deposits	+	+	+	+		+	+
	Tubular GL-3 deposits	+	+	+	+		+	+
	Glomerular endothelial cells GL-3 deposits	+	+	+	+		+	+
	Podocyte foot effacement at EM	NA	No	Segmental	Segmental		Segmental	Segmental
	Segmental sclerosis	+	-	+	-		-	-
	Global sclerosis	+	-	-	-		-	-
	Fibrosis	+	-	-	-		-	-
	Tubular atrophy	+	-	-	-		-	-
	Vasculopathy	+	-	-	-		-	-

GL-3, globotriaosylceramide; NA, not available; plus (+) sign represents presence and minus (-) sign represents absence; EM, electron microscopy.

## Data Availability

The data presented in this study are available upon request from the corresponding author.
